# Preliminary Study on the Development of a Biodegradable Functional Nasal Packing Material

**DOI:** 10.3390/polym17131878

**Published:** 2025-07-05

**Authors:** Dong Hoon Lee, EunA So, Faizan E Mustafa, Jae-ho Jeong, Bong-Kee Lee

**Affiliations:** 1 Department of Otolaryngology-Head and Neck Surgery, Chonnam National University Medical School & Hwasun Hospital, Hwasun 58128, Republic of Korea; leen3l@jnu.ac.kr; 2 Department of Microbiology, Chonnam National University Medical School, Hwasun 58128, Republic of Korea; thdmsdk6879@naver.com; 3 School of Mechanical Engineering, Chonnam National University, 77 Yongbong-ro, Buk-gu, Gwangju 61186, Republic of Korea; faizanemustafa170@gmail.com

**Keywords:** endoscopic sinus surgery, nasal packing, polyvinyl alcohol, carbon dots, chitosan, antibacterial, biocompatibility

## Abstract

Introduction: Functional endoscopic sinus surgery is commonly performed to treat paranasal sinus diseases, often necessitating nasal packing to control bleeding and aid healing. However, current materials can cause discomfort or lack adequate antibacterial properties. This study aimed to develop a biodegradable, biocompatible nasal packing material by combining polyvinyl alcohol (PVA) and carbon dots (CDs), and to evaluate its antibacterial activity and tissue compatibility. Materials and Methods: Electrospun nanofiber membranes were fabricated using PVA and biomass-derived CDs. Antibacterial efficacy of nasal packing variants (PVA, PVA-chitosan [CS], PVA-CS-CDs-1 mL, and PVA-CS-CDs-2 mL) was assessed using the Kirby–Bauer disk diffusion method against *Escherichia coli*, *Salmonella* spp., and *Staphylococcus aureus*. The in vivo biocompatibility was evaluated via histological analysis following implantation into the nasal cavity of mice. Results: All materials demonstrated antibacterial activity, with PVA-CS-CDs-2 mL showing the largest inhibition zones. Histological examination revealed minimal epithelial damage and no inflammation, with PVA-CS-CDs-2 mL yielding the most favorable tissue response. Conclusion: The PVA-CS-CDs composite demonstrates potential as a biocompatible, antibacterial nasal packing material. Further studies are warranted to validate its long-term clinical utility.

## 1. Introduction

Functional endoscopic sinus surgery (FESS) is the most commonly performed procedure for treating paranasal sinus conditions requiring surgical intervention [1,2,3]. Postoperatively, nasal packing is routinely applied to prevent bleeding, support the middle turbinate, and facilitate wound healing [1,2,3]. However, the insertion and removal of conventional nasal packing materials are often associated with patient discomfort, mucosal trauma, and secondary bleeding. In recent years, biodegradable nasal packing materials have garnered attention for their potential to reduce patient discomfort [1,2].

However, existing nasal packing materials have limitations, including reliance on animal-derived components and associated discomfort. To overcome these limitations, a novel nasal packing material was developed using polyvinyl alcohol (PVA), a biocompatible polymer widely utilized in medical, cosmetic, and pharmaceutical applications [1,4,5,6,7,8,9,10,11,12,13]. Chitosan (CS), a natural polysaccharide derived from chitin, possesses excellent biocompatibility, biodegradability, and inherent antibacterial activity. The combination of PVA and CS has been shown to enhance both mechanical and biological properties, making them suitable for biomedical applications, including nasal packing [6,7,12,13].

Carbon dots (CDs) are a novel class of carbon-based nanomaterials that exhibit unique optical properties, water solubility, low cytotoxicity, and excellent antimicrobial effects. CDs can be synthesized from various natural sources and have been increasingly utilized in biomedicine for drug delivery, imaging, and wound healing [4].

To fabricate the composite as a clinically applicable nasal packing material, electrospinning was selected over conventional film casting due to its ability to generate nanofiber membranes with properties tailored to the physiological demands of postoperative nasal healing. Unlike dense, less permeable drop-cast films, electrospun nanofiber membranes exhibit a highly porous and interconnected fibrous architecture that closely resembles the extracellular matrix (ECM) [14]. This unique structure facilitates superior oxygen permeability, breathability, and fluid absorption, which are critical for promoting mucosal recovery in the nasal cavity. Additionally, their high surface-area-to-volume ratio supports cell adhesion, proliferation, and tissue regeneration [15]. The porous structure also facilitates rapid fluid uptake and localized hemostasis by concentrating red blood cells at the wound site, thereby accelerating the coagulation process [15].

In this study, we aimed to develop a novel biodegradable and functional nasal packing material composed of PVA, CS, and CDs. Our objective was to assess the morphological, antibacterial, and histological characteristics of this composite material and to determine its potential applicability in postoperative nasal care. Furthermore, it was investigated whether incorporating CDs into PVA-based nasal packing enhances its therapeutic efficacy.

## 2. Materials and Methods

### 2.1. Fabrication of Electrospun Nanofiber Membranes

#### 2.1.1. Materials

Polyvinyl alcohol (PVA) powder (Mw = 146,000–186,000; >99% hydrolyzed), CS with a medium molecular weight (200–800 cP, 1 wt% in 1% acetic acid, 75–85% deacetylated) were obtained from Sigma-Aldrich Korea Ltd. (Seoul, Republic of Korea). Fresh apples were purchased from the local market in Gwangju, South Korea.

#### 2.1.2. Preparation of CDs

Carbon dots (CDs) were synthesized via a one-step hydrothermal carbonization process using apple peels as the carbon source, following a previously reported method with slight modifications [16,17,18]. Fresh apples were thoroughly washed; their peels were separated, oven-dried at 60 °C for 12 h, and ground into powder using a kitchen grinder. Two grams of the apple peel powder were dispersed in 50 mL of distilled water and ultrasonicated for 20 min to ensure uniform dispersion. The mixture was transferred to a 100 mL Teflon-lined stainless steel autoclave, heated at 200 °C for 12 h, and then allowed to cool naturally to room temperature. The reaction parameters were selected based on prior studies [16,17]. The resulting CD solution was filtered through a 0.22 μm membrane filter to remove large particles. Further purification was achieved by centrifugation at 12,000 rpm for 15 min to eliminate residual solids. The purified carbon dots solution had a concentration of 1 mg/mL and was stored at 4 °C until further use. The size and morphology of the synthesized CDs were previously characterized by our research group, and they were found to be spherical in shape with an average diameter of 7.36 nm [16].

#### 2.1.3. Polymer Solution Preparation for Electrospinning

An 8 wt% PVA solution was prepared by dissolving PVA powder in deionized water under magnetic stirring at room temperature (25 °C) for 1 h. The mixture was then heated to 90 °C and stirred continuously for 3 h until a clear, homogeneous solution was obtained and subsequently cooled to room temperature. Simultaneously, a 2 wt% chitosan (CS) solution was prepared by dissolving chitosan in 2% (*v*/*v*) aqueous acetic acid at 65 °C under constant stirring for 3 h. After complete dissolution, the PVA and CS solutions were blended at a 70/30 weight ratio, followed by the addition of 1 mL and 2 mL of the synthesized carbon dot (CD) solution (1 mg/mL). The final mixture was stirred at 250 rpm for 3 h to ensure uniform dispersion of all components and obtain a homogenous electrospinning solution.

#### 2.1.4. Electrospinning of Nanofiber Membranes

**Figure 1:**
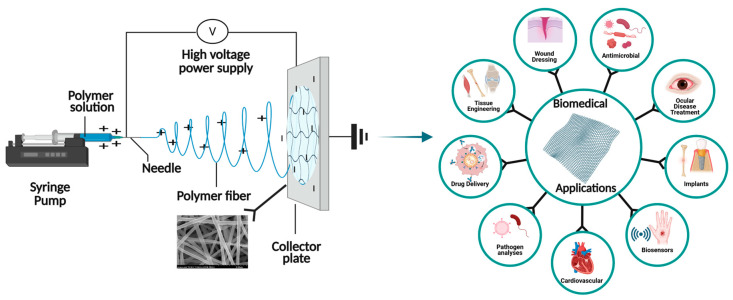
Electrospinning setup for fabricating the polyvinyl alcohol (PVA)/chitosan (CS)/carbon dots (CDs) nanofiber membrane.

**Figure 2:**
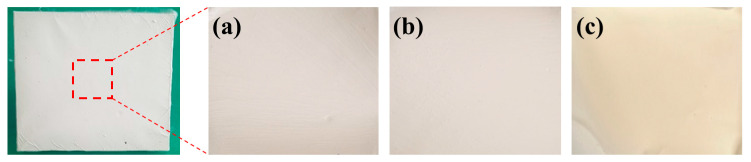
Electrospun membranes: (**a**) polyvinyl alcohol (PVA) nanofiber, (**b**) polyvinyl alcohol (PVA)/chitosan (CS) nanofiber, (**c**) polyvinyl alcohol (PVA)/chitosan (CS)/carbon dots (CDs) nanofiber.

Electrospinning was performed using a laboratory-scale setup (Figure 1) under optimized conditions. A high voltage of 13 kV was applied at the needle tip, with a feed rate of 0.3 mL/h. The needle was positioned 15 cm from the collector, where nanofibers were collected on an aluminum foil-covered metal plate. The electrospinning was carried out for 3 h at 25 °C, resulting in nanofiber membranes with a thickness of 225 ± 3.47 µm (Figure 2).

#### 2.1.5. Scanning Electron Microscope (SEM) Analysis

Nanofiber morphology and fiber diameter were examined by SEM (S-4700, Hitachi Ltd., Tokyo, Japan). Samples were mounted on bronze stubs and platinum-coated to enhance conductivity. Imaging was performed at 5.0 kV accelerating voltage and 11 mm working distance. Fiber diameters were measured using ImageJ 1.54g software, NIH, Bethesda, MD, USA.

### 2.2. Antimicrobial Susceptibility Testing

The antimicrobial activity of nasal packing materials was assessed using the Kirby–Bauer disk diffusion method on Mueller–Hinton agar (M173, HiMedia Laboratories Private Limited, Mumbai, India), following National Committee for Clinical Laboratory Standards guidelines. Packing materials were cut into uniform disks, sterilized with ultraviolet (UV) light, and placed on agar plates inoculated with bacterial suspensions.

Ampicillin (10 µg) disks served as the positive control, while sterile paper disks were used as the negative control. The test organisms included *Escherichia coli*, *Salmonella* spp., and *Staphylococcus aureus*, each prepared at concentrations of 10^6^, 10^7^, and 10^8^ colony-forming units (CFU)/mL. The experimental groups comprised positive control (ampicillin disk), negative control (paper disk), PVA, PVA–chitosan (PVA-CS), PVA-CS combined with 1 mL of CDs (PVA-CS-CDs-1 mL), and PVA-CS combined with 2 mL carbon dots (PVA-CS-CDs-2 mL). Following incubation, inhibition zones were measured to evaluate the antibacterial efficacy of each formulation.

### 2.3. Animal Experiment

#### 2.3.1. Mouse Model and Packing Procedure

Seven-week-old female C57BL/6 mice were obtained from Orient Bio Inc. (Gyeonggi-do, Seoul, Republic of Korea). All experimental procedures were approved by the Chonnam National University Animal Research Committee (CNU IACUC-H-2025-6). Nasal packing materials were standardized to 0.5 cm × 2 cm, rolled, and inserted into the right nasal cavity. The left nasal cavity remained untreated; it served as an internal control for assessing mucosal integrity.

#### 2.3.2. Histological Analysis

Mice were deeply anesthetized and perfused with saline, followed by 10% neutral buffered formalin, and decapitated. Nasal cavities were locally irrigated with the same fixative. Heads were skinned, trimmed, and immersed in formalin for 2 days, then decalcified in 10% ethylenediamine tetraacetic acid (EDTA) for 4 weeks.

**Figure 3:**
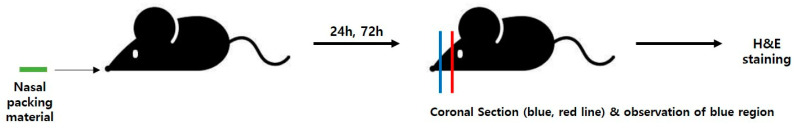
Schematic diagram of the in vivo mouse model for nasal packing evaluation. Nasal packing materials were inserted into the right nasal cavity of C57BL/6 mice. After 24 and 72 h, mice were sacrificed; heads were harvested and fixed. Serial coronal sections (5 μm) were prepared at the anatomical site marked by the blue line, following preliminary localization by the red line. Hematoxylin and eosin (H&E) staining assessed mucosal response to implanted materials.

Following decalcification, specimens were embedded in paraffin, and serial coronal sections (5 μm thick) were prepared (Figure 3). These sections were mounted on microscope slides (Fisherbrand, Waltham, MA, USA), deparaffinized, rehydrated, and stained with hematoxylin and eosin (H&E). Histological evaluation was performed using a Zeiss Axioscan 7 (Carl Zeiss AG, Jena, Germany).

## 3. Results

### 3.1. SEM Analysis

**Figure 4:**
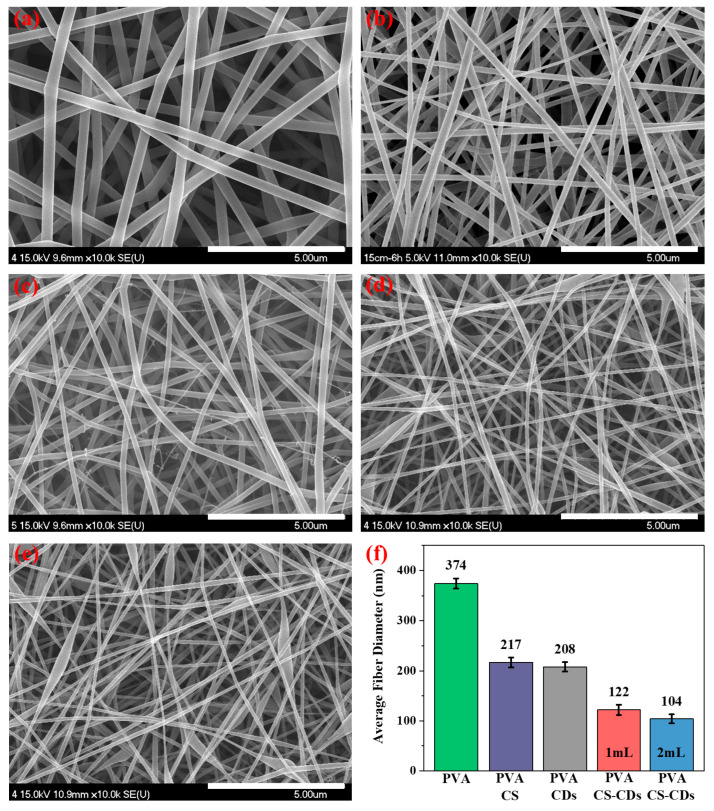
Scanning electron microscopic (SEM) images of: (**a**) Electrospun polyvinyl alcohol (PVA) fibers, (**b**) Electrospun PVA/CS fibers, (**c**) Electrospun PVA/CDs fibers, (**d**) Electrospun PVA/CS/CDs (1 mL) fibers, (**e**) Electrospun PVA/CS/CDs (2 mL) fibers, (**f**) Average fiber diameter of electrospun nano-fibers in different compositions. Data represents mean ± SD (*n* = 50). Scale bar = 5 µm (×10,000 Magnification). CDs represent carbon dots.

The surface morphology of electrospun nanofibers composed of PVA, PVA/CS, PVA/CDs, and PVA/CS/CDs is shown in Figure 4a–e. All samples exhibited uniform, bead-free, and randomly oriented fibers. The resulting nanofiber membranes formed an interconnected fibrous and porous network, which provides a supportive environment for cell proliferation, adhesion, and tissue regeneration. The average fiber diameters of each sample were quantified and are presented in Figure 4f.

Pure PVA nanofibers exhibited a smooth and uniform morphology with an average diameter of 374 ± 5.3 nm. Upon the incorporation of chitosan (CS), the fiber diameter decreased to 217 ± 4.9 nm, which can be attributed to increased solution viscosity and improved chain entanglement. Furthermore, the introduction of carbon dots (CDs) into the PVA and PVA/CS matrix led to a significant reduction in diameter. Specifically, the diameters were 208 ± 6.5 nm for PVA/CDs, 122 ± 5.4 nm for PVA/CS/CDs (1 mL CDs), and 104 ± 4.7 nm for PVA/CS/CDs (2 mL CDs), respectively.

This progressive reduction in diameter is primarily due to the enhanced ionic conductivity imparted by CDs, which promotes more efficient jet stretching under the applied electric field [19]. Additionally, the presence of CDs decreases the surface tension of the precursor solution, thereby reducing the resistance to electrostatic forces and facilitating the formation of finer fibers [20]. Notably, branched fiber morphology was observed in the PVA/CDs nanofibers sample. This phenomenon is attributed to electrospinning jet instability caused by the altered electric field distribution and increased charge density induced by the CDs [20]. These changes promote the formation of secondary jets, leading to branching behavior in which thinner fibers emerge from the primary fiber stream [21].

### 3.2. Antimicrobial Susceptibility Testing

**Figure 5:**
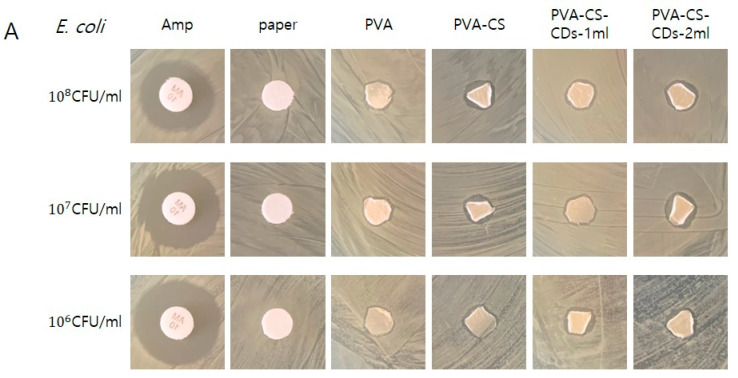

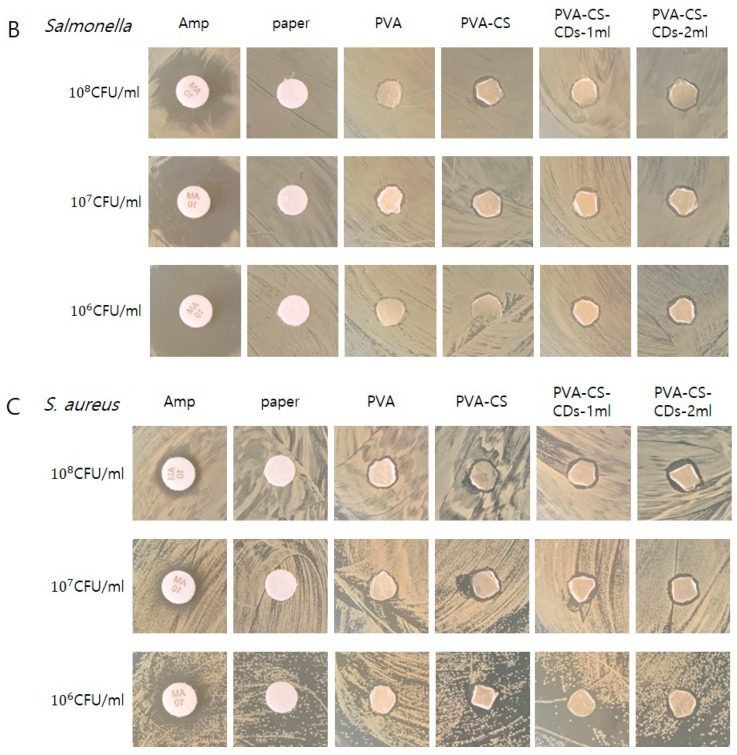
Antibacterial activity of nasal packing materials against bacterial pathogens. (**A**) *Escherichia coli*, (**B**) *Salmonella* spp., and (**C**) *Staphylococcus aureus* tested at concentrations of 10^6^, 10^7^, and 10^8^ colony-forming units (CFU)/mL by Kirby–Bauer disk diffusion. Tested materials: PVA, PVA-CS, PVA-CS-CDs-1 mL, and PVA-CS-CDs-2 mL. Ampicillin disks served as the positive control; sterile paper disks as the negative control.

All nasal packing materials tested (PVA, PVA-CS, PVA-CS-CDs-1 mL, and PVA-CS-CDs-2 mL) demonstrated antibacterial activity against *E. coli*, *Salmonella* spp., and *S. aureus* across all bacterial concentrations (10^6^, 10^7^, and 10^8^ CFU/mL) (Figure 5). Among them, the PVA-CS-CDs-2 mL group consistently exhibited the largest inhibition zones, indicating superior antibacterial efficacy, particularly under higher bacterial loads. These findings suggest a dose-dependent improvement in antibacterial performance with increasing CD content.

### 3.3. Animal Experiment

**Figure 6:**
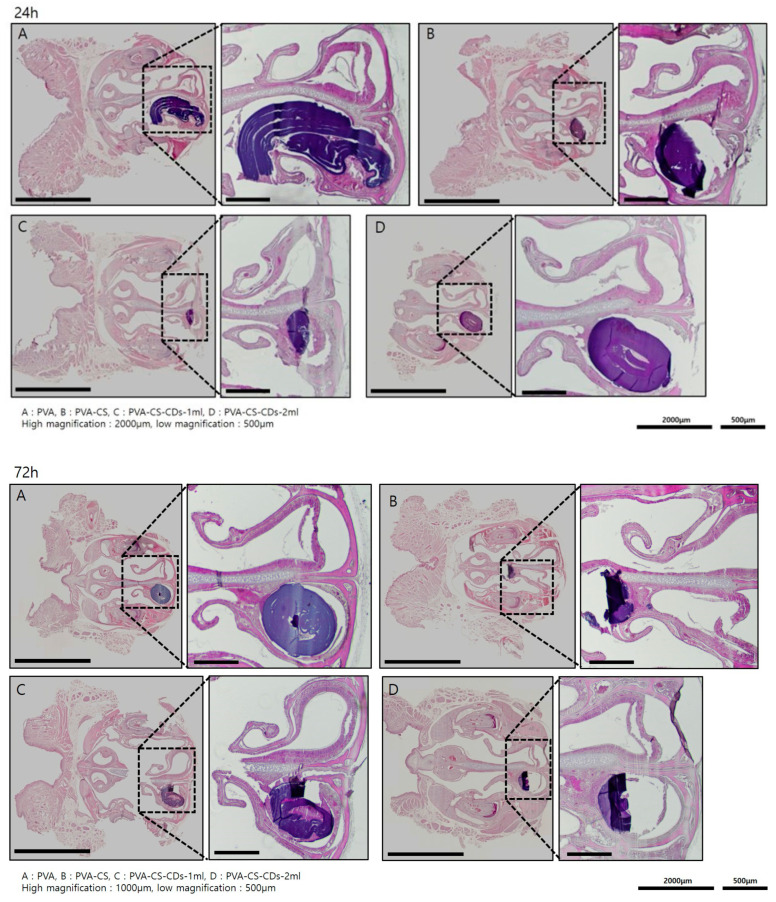
Histological evaluation of nasal mucosa after packing insertion at 24 (**upper figure**) and 72 h (**lower figure**). Hematoxylin and eosin (H&E)-stained coronal sections from mice implanted with nasal packing materials: (**A**) PVA, (**B**) PVA-CS, (**C**) PVA-CS-CDs-1 mL, and (**D**) PVA-CS-CDs-2 mL. Scale bars: 2000 μm (low magnification), 500 μm (high magnification).

Histological analysis of H&E-stained sections revealed tissue alterations in the nasal mucosa at the packing site compared to the untreated contralateral side (Figure 6), evident at both 24 and 72 h post-insertion. No inflammatory cell infiltration, vasodilation, or edema was observed. While all materials induced some epithelial damage and inflammatory response, the PVA-CS-CDs-2 mL group demonstrated relatively milder histopathological changes at 72 h. This difference suggests enhanced mucosal compatibility and reduced inflammation with increased CD incorporation.

## 4. Discussion

An ideal nasal packing material should provide hemostasis, support the middle turbinate, promote wound healing, and minimize discomfort during removal [1,2]. The present study explored the development and evaluation of a biodegradable, functional nasal packing material composed of PVA, CS, and CDs. The integration of these three components was intended to leverage their complementary properties for optimized postoperative care following FESS.

Polyvinyl alcohol (PVA) is a widely used biodegradable polymer known for its optical stability, biocompatibility, non-toxicity, and robust mechanical properties [1,4,5,6,7,8,9,10,11,12,13]. Numerous studies have demonstrated its efficacy in promoting wound healing [4,7,8,10,11,12,13]. Although commercially available PVA-based nasal packing materials exist, they are typically non-absorbable [1]. Notably, PVA’s superior mechanical strength and hydrophilic nature enhance its compatibility with other polymers, improving composite material performance [4,6,7,12,13].

Chitosan (CS) has a number of commercial and possible biomedical uses. Its intrinsic antibacterial properties are attributed to the polycationic nature of its amino groups, which interact with negatively charged microbial cell membranes, leading to cell lysis. In addition, chitosan contributes to hemostasis by promoting platelet aggregation and accelerating blood clot formation. The biocompatibility of chitosan is well documented, and its mucoadhesive properties may facilitate prolonged contact with nasal tissues, thereby improving local therapeutic efficacy [6,7,12,13].

Carbon dots (CDs) have garnered significant attention in biomedicine owing to their excellent biocompatibility, water solubility, ease of synthesis, and intrinsic antibacterial properties [4]. A CD-PVA composite film exhibits antibacterial properties and promotes wound healing in skin injuries [4], indicating the potential for improved nasal packing materials through such integration.

Consistent with previous studies, all nasal packing materials tested in this study (PVA, PVA-CS, PVA-CS-CDs-1 mL, and PVA-CS-CDs-2 mL) exhibited antibacterial activity against *E. coli*, *Salmonella* spp., and *S. aureus* across all tested concentrations [1,2,4,5,6,7,8,9,10,11,12,13]. Among these, PVA-CS-CDs composites demonstrated the most potent antibacterial effects, with the 2 mL CD formulation yielding the largest inhibition zones, suggesting a dose-dependent enhancement in antibacterial efficacy. The results suggest that the incorporation of biomass-derived CDs substantially enhances the antimicrobial properties of nasal packing materials, potentially reducing the risk of postoperative infections.

PVA, CS, and CD have been widely used in various medical applications [1,4,5,6,7,8,9,10,11,12,13]. In this study, their biocompatibility was validated by inserting the nasal packing materials into the nasal cavities of mice and confirming their safety in vivo. Histological evaluation showed that all tested formulations (PVA, PVA-CS, PVA-CS-CD-1 mL, and PVA-CS-CD-2 mL) induced minimal or no tissue damage compared to the contralateral nasal mucosa. No inflammatory cell infiltration, vasodilation, or tissue edema was observed. The PVA-CS-CDs-2 mL group exhibited the least epithelial damage, suggesting an enhanced mucosal healing potential. These findings are crucial as excessive tissue response or inflammation can lead to delayed healing or adhesion formation in the nasal cavity. Thus, the composite formulation not only provides antimicrobial protection but also ensures tissue preservation and recovery.

These findings suggest that incorporating CDs into a PVA-CS matrix may yield a biocompatible nasal packing material with robust antimicrobial efficacy and improved tissue compatibility. While this is a preliminary study, it lays the groundwork for future investigations involving mechanical property optimization, controlled drug release incorporation, and ultimately, clinical trials. Further studies are warranted to assess long-term in vivo performance and potential clinical applications.

## 5. Conclusions

This preliminary study successfully developed and evaluated a novel biodegradable nasal packing material comprising PVA, CS, and CDs. The composite exhibited potent antibacterial activity against clinically relevant bacterial pathogens and favorable mucosal compatibility in a murine model. Among the tested variants, the PVA-CS-CDs-2 mL formulation provided the most favorable antibacterial effect and histological safety. These findings support the potential of CD-integrated PVA-based nasal packing to enhance postoperative outcomes following nasal surgery. Further investigations, including long-term implantation and clinical trials, are needed to validate these benefits and explore clinical translation.

## Data Availability

The data presented in this study are available on request from the corresponding author.
